# A Fast Stereo Matching Network with Multi-Cross Attention

**DOI:** 10.3390/s21186016

**Published:** 2021-09-08

**Authors:** Ming Wei, Ming Zhu, Yi Wu, Jiaqi Sun, Jiarong Wang, Changji Liu

**Affiliations:** 1 Changchun Institute of Optics, Fine Mechanics and Physics, Chinese Academy of Sciences, Changchun 130033, China; weiming19@mails.ucas.ac.cn (M.W.); wuyi19@mails.ucas.ac.cn (Y.W.); sunjiaqi19@mails.ucas.ac.cn (J.S.); wangjiarong@cust.edu.cn (J.W.); liuchangji18@mails.ucas.ac.cn (C.L.); 2 University of Chinese Academy of Sciences, Beijing 100049, China

**Keywords:** stereo matching, depth image, computer vision, cost volume, disparity regression

## Abstract

Stereo matching networks based on deep learning are widely developed and can obtain excellent disparity estimation. We present a new end-to-end fast deep learning stereo matching network in this work that aims to determine the corresponding disparity from two stereo image pairs. We extract the characteristics of the low-resolution feature images using the stacked hourglass structure feature extractor and build a multi-level detailed cost volume. We also use the edge of the left image to guide disparity optimization and sub-sample with the low-resolution data, ensuring excellent accuracy and speed at the same time. Furthermore, we design a multi-cross attention model for binocular stereo matching to improve the matching accuracy and achieve end-to-end disparity regression effectively. We evaluate our network on Scene Flow, KITTI2012, and KITTI2015 datasets, and the experimental results show that the speed and accuracy of our method are excellent.

## 1. Introduction

The binocular camera plays a significant role in autonomous driving, target detection, and other fields. It has a series of advantages such as a much lower price than LIDAR, better performance, and fewer errors [1,2]. We can use the binocular camera to achieve excellent depth estimation from a pair of corrected left and right images. Then we can estimate the 3D geometry to carry out the next stage of 3D target recognition, 3D reconstruction, and other tasks [3]. The core of stereo vision is stereo matching technology, which is a classic in computer vision. It is a difficult and vital step to estimate the depth by calculating the relationship between the left and right images to pixels [4].

The purpose of stereo matching is to find the corresponding pixels from the binocular images [5]. The pixel point (x, y) is in the left image; the same pixel point is (x − d, y) in the right. Through disparity d, the depth D of the pixel is fB/d, where f is the focal length of the camera and B is the baseline distance between the center of two cameras [6,7].

For many years, people have applied deep learning to stereo matching [8]. Using the network to learn parameters and predict the disparity image, people continue to find and solve problems in stereo matching [9]. In stereo vision, there is sometimes the loss of texture details, periodically repeated features, and other conditions in flat areas because of the effects of camera shooting and ambient light. If not effectively processed, it is easy to produce wrong matching [10,11]. At the same time, to ensure the accuracy of the case, we should reduce the complexity of the implementation and calculation as far as possible.

**Figure 1:**
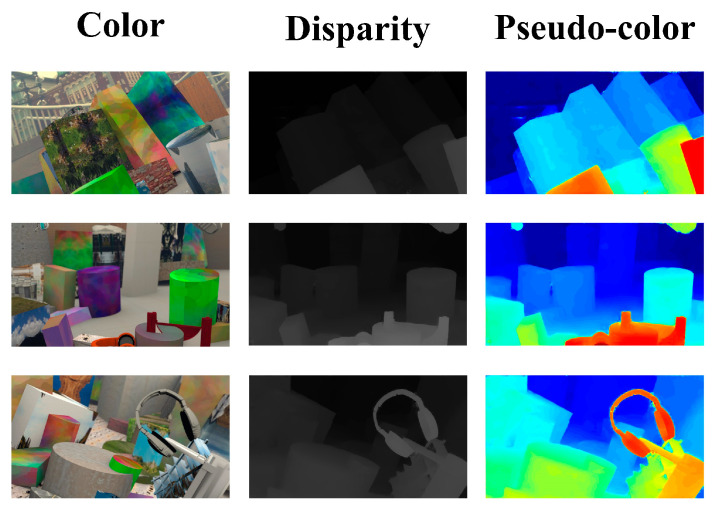
Visualization of disparity prediction results using our network on Scene Flow dataset. The first column is RGB images, the second column is disparity images estimated by our method, and the last column is the pseudo-color disparity images for showing them more clearly.

**Figure 2:**
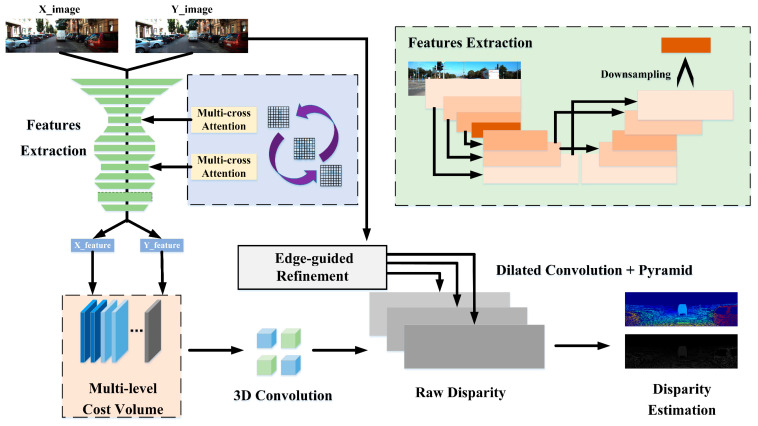
Our network structure. It consists of a feature extraction layer with multi-cross attention, multi-level cost volume layer, 3D convolutional layer, and edge-guided optimization network. First, the input is the left and right matching image pairs with the correction of the input pole line. The next layer is a stacked hourglass feature extractor that provides a down-samping low-resolution feature image. The features of the binocular images are fully extracted by this layer with shared weight [32], and two corresponding feature images of the binocular are obtained. At the same time, the purple part is the multi-cross attention module, which is placed before the convolutional layer of the feature extractor to improve the ability. Then we combine the left and the right feature images into a whole through a multi-level cost volume layer. The two feature images form a multi-level cost volume of 96 channels to improve the accuracy. Next, the raw disparity predictions are regressed by the cost volume through the 3D convolutional layer. Finally, we optimize the efficient disparity image by the edge guidance of the RGB image through the optimization network.

In our research, we adopt some ideas from GA-Net [12] and Stereo-Net [13]. However, a large amount of 3D convolution and complex optimization in GA-net lead to slow speed, and the parallax estimation of Stereo-Net is not effective enough to know the rich 3D information. By contrast, our network is faster and more accurate. As shown in Figure 1 and Figure 2, we design an end-to-end stereo matching network (MCA-Net) and achieve effective results. Our network advantages are as follows:

(1) We designed a multi-cross attention module and added it to the feature extractor, and we increased the ability of feature extraction and improved the matching accuracy.
(2) Compared to the single-level construction method, we used a multi-level cost volume construction and achieved a better disparity estimation.
(3) Due to the high complexity and the time consumption of 3D convolutions, we improved the feature extraction network of the stacked hourglass. We used low-resolution features to construct the cost volume, which reduces the amount of computing data of the 3D convolution network and improves the speed significantly.

## 2. Related Work

Many traditional classical stereo matching methods and stereo matching networks based on deep learning have been proposed for many years [13,14]. The stereo matching networks include the two-step stereo matching network and the end-to-end matching network without post-processing.

Two-step network: The researchers worked on the same idea as the steps of traditional methods to separate stereo matching from parallax optimization and added post-processing after matching by the neural network [15]. Classic stereo matching network MC-CNN [16] uses pixel matching and the shared weight dual network to extract image features to predict another view similar to the corresponding image block at the center of each pixel. We obtain the estimated disparity images after aggregating and calculating the cost difference. Content CNN [17] is similar to MC-CNN [16], which only loses a small amount of accuracy but can speed up the calculation of binocular disparity images. Traditional SGM [18] is used in practice widely. We obtain excellent disparity estimation by semi-global aggregation. Therefore, based on traditional SGM [18], SGM-Net [19] uses a convolutional neural network to calculate penalty terms and achieves a better effect. On the other hand, some researchers proposed new post-processing methods. TDSR [20] uses hierarchical segmentation by the waterfall and robust regression models to propagating disparity values as a post-processing step for any stereo-matching algorithm. Post-processing is a necessary step, but these networks have almost been replaced by end-to-end networks.

End-to-end network: Because there are many steps in a two-step network and the method is complicated, many end-to-end deep learning matching networks have since sprung up. These networks add post-processing steps to the network, as does our network. For the first time, GC-Net [21] extended 3D features to 4D features and extracted context information from matching cost volume by 3D convolution on the disparity dimension without additional post-processing or regularization operations, and it obtained end-to-end sub-pixel accuracy estimation. Based on the cost volume, the Spatial Pyramid Pooling module and stacked hourglass 3D convolutional layer are added into PSM-Net [22] to improve the prediction effect. There were many efforts and ideas proposed by researchers to improve accuracy. Edge-Stereo [23] breaks the current situation that the end-to-end binomial vision network extended on the disparity aggregation network and proposed a new network that uses the contextual information pyramid and multi-task learning for prediction. Seg-Stereo [24] adds a semantic segmentation network to integrate the segmented semantic features with feature graphs, making full use of image structural information to improve the prediction accuracy of disparity images. They performed well in both unsupervised and supervised modes. Deep Pruner [25] is different from other complete cost spaces constructed from zero to the maximum disparity range. Patch Match [26] closes the matching search range of each pixel according to the preliminary disparity calculated in advance. In this way, we obtain a sparse cost space using the image-guided optimization module to improve the performance. The cost space obtained through adaptive pruning contains fewer parameters and is more efficient in cost aggregation. GA-Net [12] takes the weight of SGM [18] as part of the network prediction, learns the custom parameters, and uses the optimized aggregation network to improve the accuracy of disparity estimation. CSPN [27] can not only predict depth but also complete depth. We obtain more accurate and precise results by optimizing the relationship between each point and the prediction of adjacent points. Based on the linear model of the cyclic neural network, SDR [28] refines the disparity plane by learning the correlation matrix between adjacent pixels and using global and local two-level optimization. SSPCV-NET [29] improves the aggregation cost by using multi-scale feature information to form a pyramid cost in the end-to-end neural network. With the development of self-supervised learning, PV Stereo [30] builds multi-scale cost measurements and updates disparity estimation at high resolution by using cyclic units and generates reliable semi-density disparity images that supervise training and perform self-supervised stereo matching.

In stereo matching methods, the difficult difficulty is the mutual exclusion of accuracy and speed. With the 3D convolution was proposed by GC-Net, most stereo matching networks have used it to achieve better disparity estimation. Generally, the 3D convolution layers help to obtain a more detailed feature extraction. However, it has paid a high time cost, and the real-time cannot be satisfied. Two ideas are most common to improve the running speed: one is to find an alternative to 3D convolution, and the other is to reduce the resolution to reduce the calculation.

AA-Net [31] uses the same scale aggregation module (ISA) and the cross-scale aggregation module (CSA) to replace the 3D convolution commonly used in the stereo matching model. It uses the new multi-scale 2D convolutions instead of 3D convolution to reduce dimensions and improve speed. What is more, Stereo-Net [13] reduced the calculation amount and the running time by reducing the input resolution. It has a lightweight structure, fewer network parameters, and less training time. The relatively rough disparity uses the low-resolution input after the sub-sampling, and the residual network is graded and optimized by adding the pixel information of the left and right original images. We combined the two methods and put forward the new method to balance between precision and speed, reducing the number of 3D convolution layers as far as possible. We used the multi-cross attention module to extract features better and the multi-level cost volume to integrate features better. At the same time, we reduced the resolution of the input features and used edge-guided refinement to improve resolution and obtain better results without more 3D convolution layers. Compared with other networks, our network has advantages in the balance between precision and speed.

## 3. Network Architecture

In a neural network, the feature extraction layer can obtain information from the original image and is a vital step in network structure. The more abundant features, the better the effect of network learning will be. The stackable hourglass feature extractor in the classical Stereo matching network GA-Net [12] is effective. However, due to the complexity of its network structure, although it maintains a high accuracy, its speed is slow and cannot meet the demand of real-time performance. The other classical Stereo-Net [13] is a real-time high-speed matching network. The problem is that the speed of 3D convolution is low and can be solved by using guided up-sampling of low-resolution disparity images effectively. However, it costs a loss of precision. As shown in Figure 2, we built an end-to-end stereo matching network. The input and output are the RGB images and the disparity images in the network. We set the stacked hourglass structure for feature extraction, and the down-sampling can improve the speed of operation. The multi-cross attention and the multi-level cost volume improve accuracy and offset the accuracy loss caused by sub-sampling to obtain better disparity estimation.

### 3.1. Features Extraction

We used a stacked hourglass structure as the feature extractor. With the residual network proposed, many feature extraction networks are made of multiple convolutional layers. Compared with the ordinary network, the residual network has a promotion and causes less loss of information. More information can be extracted through it. Thus, we stacked the hourglass structure to make full use of the multi-scale features of different sizes and different channel convolution kernels. It is utilized to extract characteristic information of cyclical ways. At the same time, we connected the structure. We attached the copy of the same size and fusion to make full use of contexts, which increases the complexity and obtains better scale information.

Our stacked hourglass feature extractor is shown in the green box in the upper right corner of Figure 2. The image goes through layer-by-layer 2D convolution, and the alternating process of down-sampling and up-sampling is carried out to form the shape of an hourglass and stacks together. The number of channels also changes regularly with the size in this process. The same number of channels and the similar-sized features at the upper and lower ends of the hourglass are added together to form a residual structure. Finally, a low-resolution feature image is sampled after completing feature extraction of the image to realize low-resolution input of 3D convolution.

### 3.2. Multi-Cross Attention Module

**Figure 3:**
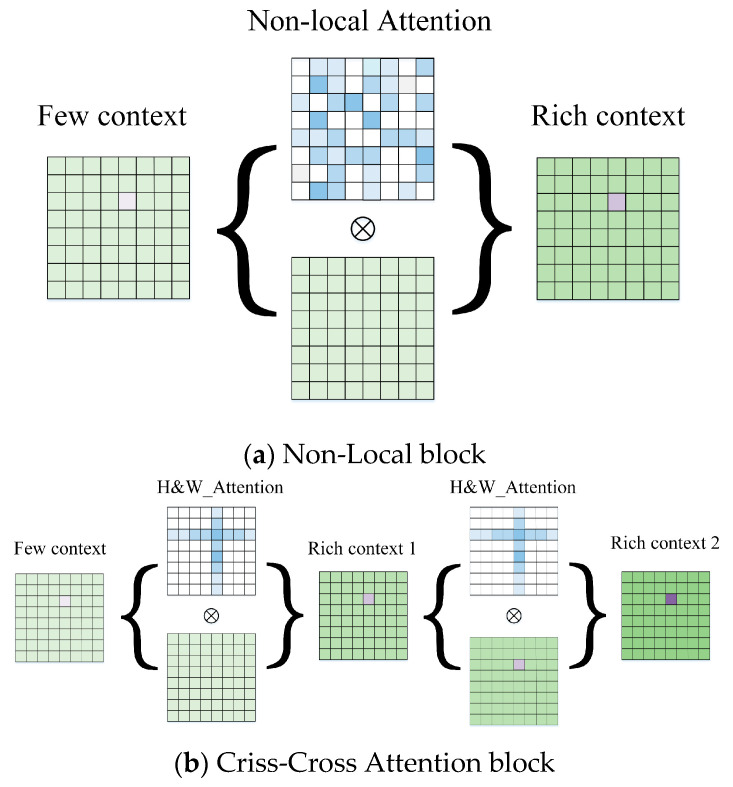

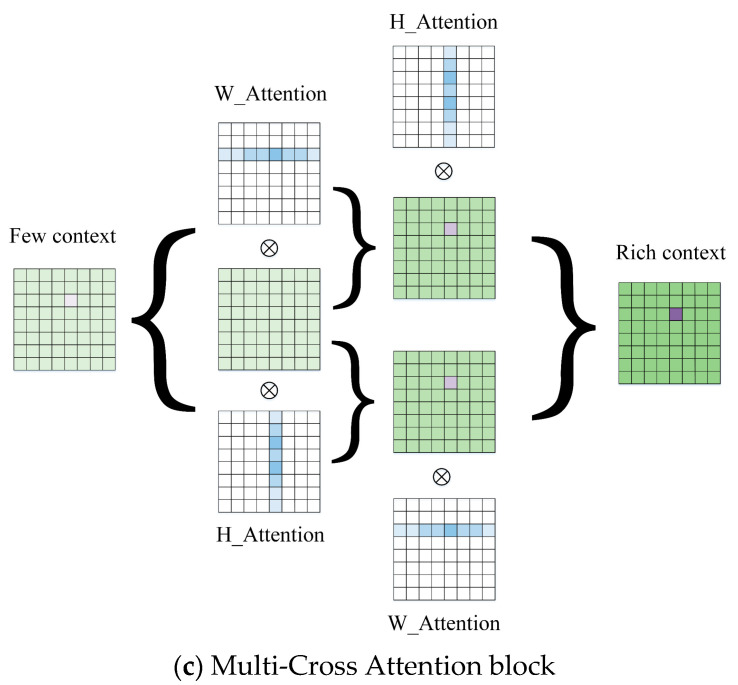
Diagram of three approaches to attentional context aggregation. The white and blue grids are different attention modules. The green grids are original feature images that changed through the attention modules. (**a**) A common non-local block with a global feature aggregation [36]. For each pixel, the aggregated result is obtained through intensive mapping with different weight allocations. (**b**) A Criss-cross Attention block [35]. The different weights are assigned in the horizontal and vertical directions serially. (**c**) A Multi-Cross Attention block that is different from (**b**). We adopted the structure in horizontal and vertical directions in parallel and separately to aggregate features more adequately. At the same time, we retained the residual connection to achieve a better feature aggregation effect.

The attention module works well for feature extraction [33,34]. In order to improve the speed, our network uses low-resolution feature image input with eight times down-sampling, which will lead to a loss of precision. Therefore, we designed a multi-cross attention network to improve the capability of the feature extractor adequately. As mentioned in the CC-Net [35] literature, the cross-concern module for each pixel can collect the context information of all pixels on its cross path, as shown in Figure 3. With further looping operations, each pixel can eventually capture the image dependencies.

**Figure 4:**
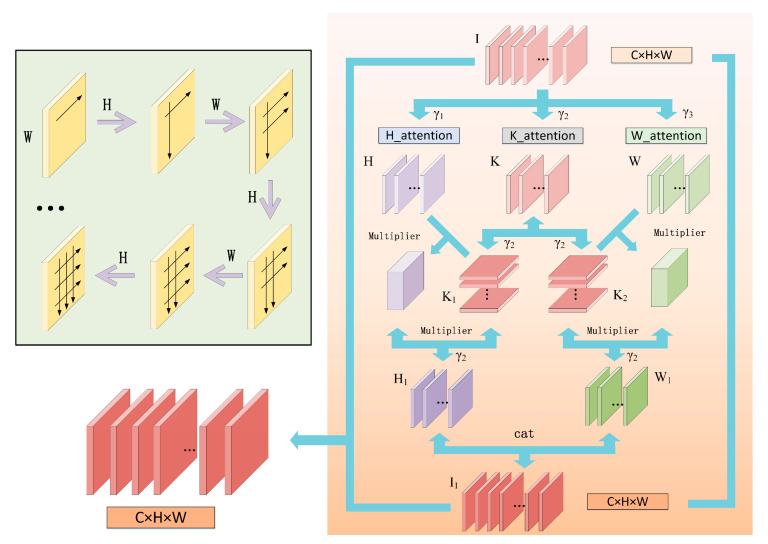
Our Multi-Cross attention module. We extracted features deeply in horizontal and vertical direction multi-cross. We set the “H_attention” as the vertical direction, the “W_attention” as the horizontal direction, and the “K_attention” as the auxiliary branch. The input eigenvector has height H, width W, and the number of channels C. With the gradual enrichment of cross operations, the feature extraction is strengthened fully, and the superiority of attention weight is gradually embodied. Finally, an efficient attention module is constructed with several multipliers and fusion operations in horizontal and vertical directions. This process is illustrated in the upper left block diagram. The final output is the same size as the input.

For the stereo matching process, we prefer the original information, because it is closer to the real. Therefore, we designed multi-cross attention that is more suitable for the matching network. Due to the pole that has been corrected before the stereo matching, the matching pixels should be on the same horizontal line. At the same time, we still needed the vertical features to integrate the context information fully because of possible errors. Therefore, we used the horizontal and vertical intersecting attention modules to deepen the extraction of detailed features. We extracted feature information several times through multiple cycles in parallel, which is different from the sequential connection of CC-Net [35]. We found the Multi-Cross Attention module does not reduce computational effort compared to the Criss-Cross Attention module and showed the experimental results in the later chapters that the speed of the two attention modules is similar. However, in terms of structure, we expanded a new branch horizontally and replaced serial with the parallel process, which achieved better results under the same time consumption. The network structure is more concise and maintains the original context of the association. The structure of our network is shown in Figure 4.

As shown in Figure 4, we construct a lightweight attention module that is memory-friendly. “I is the input of the attention module, “K” is the auxiliary branch, “H ” is the vertical branch, and “W” is the horizontal branch. The input of the module $I\in {ℜ}^{C\times H\times W}$ is regularized dimension to $I^{'}\in {ℜ}^{C_{\gamma }\times H\times W}$ by convolution, where γ is the co-efficient used for dimension reduction. There are three branches, $H^{i}\in {ℜ}^{C_{\gamma 1}\times H\times W}$, $K^{i}\in {ℜ}^{C_{\gamma 2}\times H\times W}$, and $W^{i}\in {ℜ}^{C_{\gamma 3}\times H\times W}$, where γ1=γ3. K is split and transposed into $K_{1}\in {ℜ}^{W\times C_{\gamma 2}\times H}$ and $K_{2}\in {ℜ}^{H\times C_{\gamma 2}\times W}$. Then, we integrate $H_{1}^{i}$ and $W_{1}^{i}$ by similar matrix multiplication as follows (Equation (1)):

(1) $$\begin{array}{l} H_{1}^{i}\langle {ℜ}^{W\times H\times H}\rangle =H^{i}\langle {ℜ}^{C_{\gamma 1}\times H\times W}\rangle \times K_{1}\langle {ℜ}^{W\times C_{\gamma 2}\times H}\rangle \infty \\ W_{1}^{i}\langle {ℜ}^{H\times W\times W}\rangle =W^{i}\langle {ℜ}^{C_{\gamma 3}\times H\times W}\rangle \times K_{1}\langle {ℜ}^{W\times C_{\gamma 2}\times H}\rangle \end{array}$$

where $H_{1}^{i}\langle {ℜ}^{W\times H\times H}\rangle$ is the resulting value of the first vertical branch of layer i whose size is W×H×H. At the same time, $H^{i}\langle {ℜ}^{C_{\gamma 1}\times H\times W}\rangle$ is the original value of the first vertical branch of layer i whose size is $C_{\gamma 1}\times H\times W$ and $K_{1}\langle {ℜ}^{W\times C_{\gamma 2}\times H}\rangle$ is the original value of the first auxiliary branch of layer i whose size is $W\times C_{\gamma 2}\times H$. The W branch is the same as the H branch.

Continuing the deep processing, we obtained the horizontal and vertical characteristics of the deep layer at i=1,…,s. And s is the number of levels; we chose s=1. Finally, the characteristics of the two branches will increase dimension to the size of the original. We received the final characteristics of attention weighting parameters $I_{1}\in {ℜ}^{C\times H\times W}$ for feature extraction.

### 3.3. Multi-Level Cost Volume

**Figure 5:**
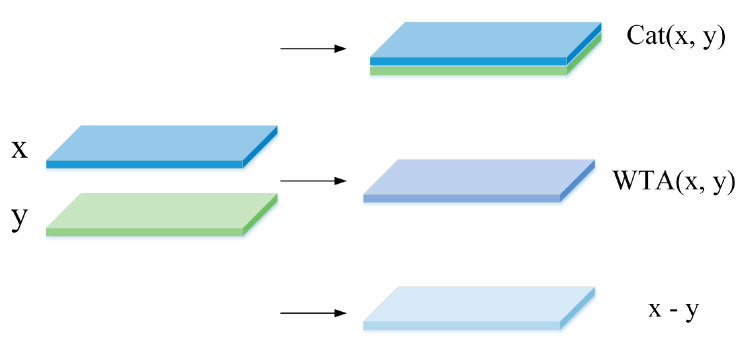
Three single ways to build cost volume. x and y features are used for channel connections, winner-take-all, and difference fusion.

In the previous step, the feature images of the binocular image pairs can be obtained. Because they are low-resolution features after the sub-sampling, the time consumed in calculation is reduced. Next, we built the cost volume to fuse the left and the right feature images extracted from the twin network. As shown in Figure 5, some other networks use single feature combinations to connect the x, y characteristics. We can find the context information fully, where x and y represent the features extracted from the left and right images. Three build methods are shown from top to bottom in Figure 5. The first one is to connect x and y directly according to the channel to get the simple fusion of the two eigenvectors. This method is simple to implement and lacks less information. The second is to use the winner-takes-all idea. The minimum disparity of Euclidean distance between two eigenvectors is selected. To a certain extent, this approach can be close to the optimal result, but the fault tolerance is very low, and some original information will be lost. The last option uses the uniqueness of the stereo matching network and only finds the disparity deviation in an image just along the polar. Therefore, we obtain more accurate disparity information by constructing the cost volume using x − y from the left and right feature images after learning the weights by sharing the twin networks.

**Figure 6:**
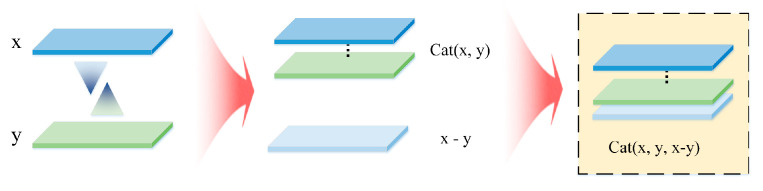
Our Multi-Level Cost Volume. For the original information, there is a limit to the distribution method of retaining and removing. Through the experiment in the following chapters, we chose the construction form in the yellow box on the right of the figure to build a multi-level cost volume by combining the original x and y features and the differential fusion.

As mentioned in other research, asymmetry representation is usually well-realized, so we used the multi-level information to construct the multi-level cost volume. By combining the two effective construction modes, the context information is not lost and enriched, and the prior knowledge of stereo matching is fully utilized to get closer to the optimal disparity information, as is shown in Figure 6.

### 3.4. 3D Convolution

3D Convolution plays a vital role in stereo matching networks. It can directly learn features of 4D cost volume, effectively avoiding the loss of information [36,37,38]. However, 3D convolutions cost a lot and take a lot of time. Therefore, we tried to minimize the use of 3D convolutions and used the above multi-level cost volume construction to reduce the loss of information instead of using more 3D convolutions. Our network only uses six 3D convolutions. We ensured an excellent effect, reduced the consumption of computation, and improved the speed.

### 3.5. Edge-Guided Refinement

**Figure 7:**
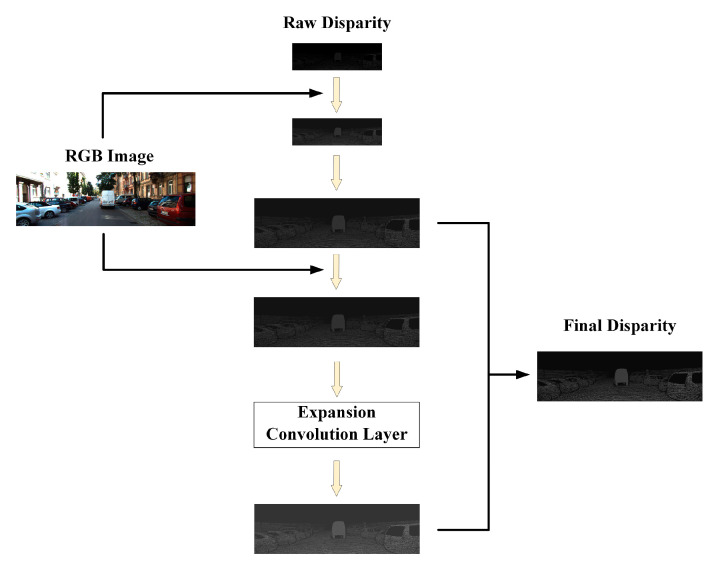
Our edge-guided refinement network. The RGB image is used to guide the 2D convolution process of the raw disparity image with two layers, and the expansion convolution is used to enhance the fusion effect. In this way, the fine disparity image is obtained in the end.

An effective disparity optimization method is proposed in Stereo-Net [13]. The authors fused the estimated low-resolution depth image with the original high-resolution color image and improved the resolution of the disparity image through multiple guidance. As a reference, the disparity image is also optimized by edge guidance in our network. As shown in Figure 7, through the RGB images, we added detailed information to low-resolution images layer by layer and added or subtracted inappropriate predicted values from rough disparity images to achieve the modification of detailed information and texture information. The disparity effect is improved.

In the refinement network, we input the initial rough disparity image outputted by 3D convolution. We needed to realize up-sampling through guided optimization to change the size of the disparity image to the size of the original image. During this process, the expansion convolution and up-sampling overlap are used to form a pyramid. Different scales of the original image are used to recover the information of different scales of the prediction graph. The expansion co-efficient is set as 1, 2, 4, 8, 1, 1, without batch normalization or activation. The output disparity is fused with the originally predicted disparity to improve the effect gradually and restore the high-frequency details.

After 3D convolutions, we obtained the initial disparity images $P^{'}\in {ℜ}^{1\times H\times W}$ whose size is 1×H×W. We refined the edges of the initial disparity images by guiding the RGB image $I\in {ℜ}^{3\times H\times W}$ whose size is 3×H×W. The pixels in the detailed disparity images $P\in {ℜ}^{1\times H\times W}$ were obtained after refining by

(2) $$P(x_{i},y_{i})=\psi (P^{'}(x_{i},y_{i}),I(x_{i},y_{i}))$$

where ψ is the fusion operation of images with two different numbers of channels. The results are better than the initial disparity images with iterations. $x_{i}$ and $y_{i}$ are the coordinate value of pixels in the image pairs.

### 3.6. Loss Function

We trained our deep learning network with smooth Huber loss (L1 loss) in a fully supervised manner. Smoothed L1 loss is robust in the position of disparity discontinuity. Compared with L2 loss, Smoothed L1 loss can reduce the noisy sensitivity and weaken the influence of outliers effectively. It is used as the basis for loss in deep learning networks widely. Smooth L1 loss is as follows:

(3) $$\mathrm{SmoothL}1=\{\begin{array}{cc} 0.5x^{2} & \left|x\right|<1\\ \left|x\right|-0.5 & \left|x\right|\geq 1 \end{array}$$

The loss function of each pixel is as follows:

(4) $$L_{i}=\sum_{k=1}^{k}SmoothL1(\gamma (d_{i}^{k}-{\hat{d}}_{i}))$$

where γ is the robust co-efficient, i is the serial number, and k is the number of iterations of disparity optimization. The larger k is, the more times of guiding optimization are, and the more detailed the disparity is. $d_{i}^{k}$ is the predicted disparity obtained after the disparity optimization, and ${\hat{d}}_{i}$ is the ground truth disparity corresponding to the same pixel of $d_{i}^{k}$. The difference value of the two is super-placed by the robust co-efficient to form the whole loss function. Based on this, we continued to iterate and finally find the optimal disparity estimation.

## 4. Experimental Evaluation

In this section, we test our performance on different data sets. Quantitative and qualitative test results are used to measure the learning ability of our algorithm. We introduce our experimental data platform in 4.1. In 4.2, we conduct ablation studies on our multi-level cost volume and multi-cross attention modules. We use the direct training on KITTI2015 in this section. In 4.3, we evaluate the accuracy and speed of our network on Scene Flow, KITTI2012, and KITTI2015, respectively, by comparing other networks.

### 4.1. Dataset and Setup

We implemented our approach in Pytorch and using Adam (β1 = 0.9, β2 = 0.999) as the optimizer. Pytorch is a deep learning framework. Adam is an optimization algorithm that can iteratively update the weight of a neural network based on the training data. We trained our model on 1 NVIDIA GPU with a batch size of 2. The learning rate starts at 0.001. When finetuning the model, the learning rate is 0.0001 after 300 epochs. For all datasets, the input images are normalized with ImageNet mean and standard deviation statistics. We kept these hardware parameters and software parameters of each training and test experiment consistent and comparable with each other. We used random color augmentation and vertical flipping and set the maximum disparity as 192 pixels. 

Scene Flow [39]: A large-scale synthetic dataset containing three sub-sets, FlyingThings3D, Monkaa, and Driving; contains everyday objects flying along random 3D paths, animated short films, and vehicular driving images similar to the KITTI dataset. Data sets provide a complete disparity image as ground truth. There are 35,454 training images and 4370 test images in the data set, H = 540, and W = 960. 

KITTI2012 [40]: A small dataset of real-world street-view related to automatic driving obtained by lidar. It contains 194 pairs of stereo images with ground truth for training and 195 pairs of stereo images without ground truth for testing, H = 376, and W = 1240. We further divided the 194 pairs of training set data into 160 pairs for training and 34 pairs for validation to evaluate our training effectiveness.

KITTI2015 [41]: KITTI2015 is similar to KITTI2012 and is also a small data set of real-world street views obtained by lidar related to autonomous driving. The dataset contains 200 pairs of stereo images with ground truth that can be used for training and 200 pairs of stereo images without ground truth for testing, H = 376, and W = 1240. We further divided the 200 pairs of training set data into 160 pairs for training and 40 pairs for verification to evaluate the effectiveness of our training. Due to the small amount of data, we first used Scene Flow to train ten epochs and then used it as a pre-training model to finetune.

### 4.2. Ablation Experiments

In order to test the effectiveness of our multi-level cost volume, we proposed an ablation study to compare the effects of conventional construction methods and our construction methods on network results to prove our design choice. It is a vital idea for us. We did not use the pre-training model and directly trained KITTI2015 without checkpoints. Small data sets tend to over-fit, so using small data sets can effectively find the problem of over-fitting synchronously. At the same time, we can quickly find whether the network is effective at the initial stage of the experiment. We can quickly judge and analyze the model through the first over-fitting location without further adjusting the learning rate. The best experimental results of each network are chosen as the final results for mutual comparison. In addition, we did not use low-resolution inputs to compare more obviously in the ablation experiment. Therefore, our results in this section are slower but more accurate than the experimental results in the following.

#### 4.2.1. Multi-Level Cost Volume

We only changed the dimension and did not add other efficient modules in the experiment. As shown in Table 1, there are four ways to construct cost volume. Dimension 32 (MCA Net-32) is x − y. Dimension 64 (MCA Net-64) is x and y. Dimension 96 (MCA Net-96) is x, y and x − y. Dimension 128 (MCA Net-128) is x, y, x − y, and x + y. The over-fitting position is the number of epochs in which the over-fitting phenomenon occurs for the first time. EPE is the mean disparity error in pixels. Avg error rate is the average percentage of the pixel whose EPE is more than one or three pixels. Run-time is the running time of a single variable under our keep-consistent setting.

**Figure 8:**
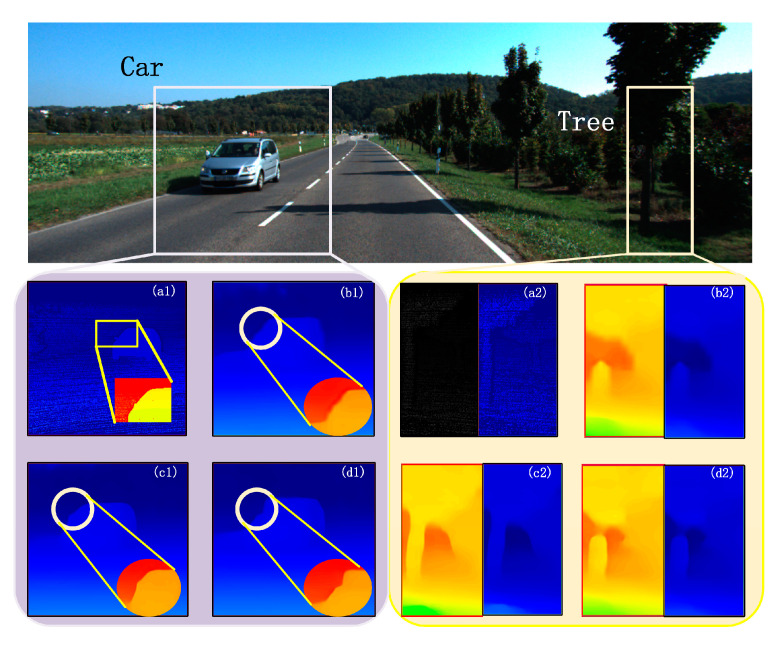
The rendering of the multi-level cost volume ablation experiment on KITTI2015. (**a1**,**a2**) are the ground truth, (**b1**,**b2**) are the result of the prediction of cost volume constructed in 32 dimensions, and (**c1**,**c2**) are in 96 dimensions, (**d1**,**d2**) are in 128 dimensions. As you can see, for the car in the left box, the shape in (**b1**,**d1**) is not fitting, and the car in (**c1**) is the similar shape. For the trunk in the right box, there are still unclear edges and target truncations in (**b2**,**d2**), the shape that best fits the true depth image in (**c2**).

The experiment in Table 1 and Figure 8 shows that training KITTI2015 using the 32-dimensional construction method has no effect directly or temporarily, and it is weak in extracting features from a small dataset. If there is a lack of a pre-training model, the feature extraction ability is missing. The structure of x − y discards a lot of effective information, so it makes it impossible to get results. Although there is only less loss in running time, the 64-dimensional construction quickly reaches over-fit and produces worse results. The 128-dimensional construction not only slows downtime but also increases EPE and Avg Error Rate values. Therefore, the 96-dimensional construction gives the best results with only 0.08s of extra time. Compared to the ordinary structure, its EPE decreased by about 0.3%, and Avg Error Rate decreased by about 2.88% in all regions. Its EPE decreased by about 0.24%, and Avg Error Rate decreased by about 1.96% in non-occluded regions. At the same time, the result shows that too many dimensions can weaken the effect. Our network builds the 96-dimensions cost volume that increases contextual information without bringing heavy redundancy and increasing the burden of the network.

#### 4.2.2. Multi-Cross Attention Module

We set a single-variable ablation method to test the effectiveness of our multi-cross attention module. We used the network without adding multi-level cost volume to conduct experiments on the network structure with and without adding multi-cross attention module. In the same way as the previous experiment, we judged the location of over-fitting and selected the best final results for comparison.

**Figure 9:**
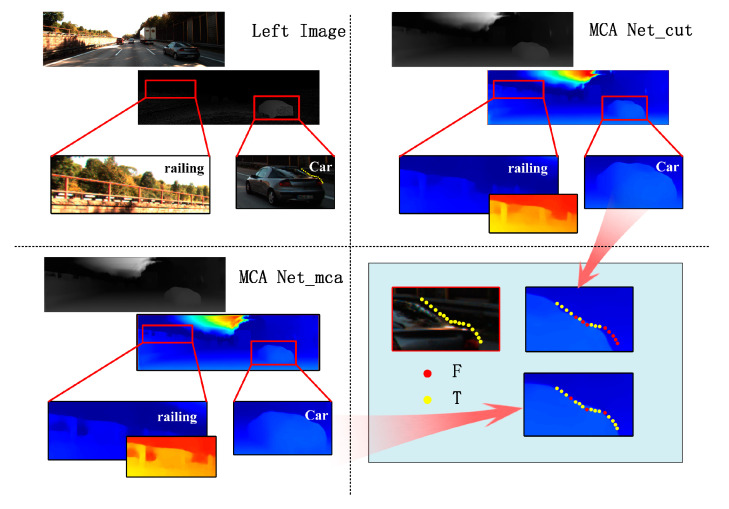
The results of the multi-cross attention module ablation experiment on KITTI2015. After adding the MCA module, the edge details of the railing have been added, and the background and foreground have become more distinct. At the same time, the shape radian of the car on the right side is obtained more obviously. The yellow points are right points as well as the red points are error points. The number of red points decreases after adding our module.

The experiment in Table 2 and Figure 9 shows that our network can effectively enhance the ability of convolution to extract features. “-cut” means the model without any attention module. “-cca” means an attentional module in the original article, which gets poor results. The run-time consumption is only less than 0.2% with our attention module, but the results reduced the EPE by about 24% and the Avg Error Rate by about 19% than before on KITTI2015.

### 4.3. Results of Experiments

Scene Flow: There is a large amount of data in Scene Flow, and there is usually no over-fitting problem in it. Therefore, the results of direct training of 10 epochs are compared here with other networks. The results are shown in Table 3. Our network has a smaller number of 3D convolutions than GA-NET-7, and the error rate is reduced by 0.79%. The Avg Error Rate of our network is only 0.31% lower than GA-NET-11, but the number of 3D convolutions is four fewer. We reduced the burden of the network effectively. Compared to Stereo-Net, PSM-Nest, and GC-Net, the accuracy is significantly higher. 

KITTI2012 and KITTI2015: There are only 160 pieces of training data here, so after training the Scene Flow ten times, we took the weight as the pre-training model, fine-tuned KITTI2015 and KITTI2012, and compared the obtained results with other network results. The results are shown in Table 4. The error rate is not at a minimum, but our network uses the minimum amount of 3D convolution, and the effect is close to that of a network with a lot of 3D convolution with less running time. Compared with Stereo-Net, the Avg Error Rate is reduced significantly. Compared to GC-Net, our network not only reduces the number of 3D convolutions by more than a third but also reduces the time by half. Compared to MC-CNN, our network is faster and better. Although the accuracy of our network decreased by approximately less than 0.2%, the speed increased by half compared to GA-Net-15 and 70% compared to SSPCV-Net. 

The experiment in Table 3 and Table 4 shows that our network can keep the accuracy accurate and ensure the speed is efficient.

**Figure 10:**
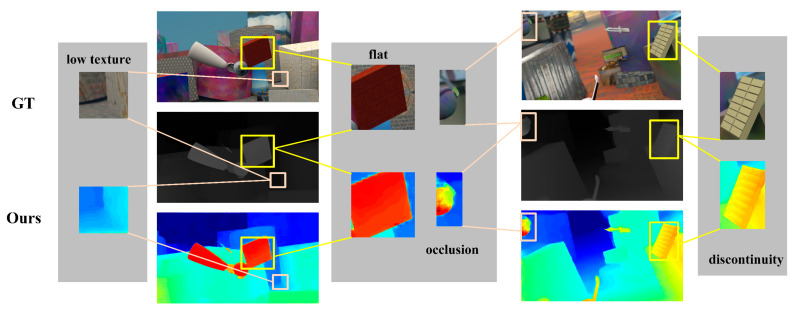
The results for discontinuity, flat, occlusion, and low texture regions on Scene Flow.

Figure 10 shows that our network works well for a wide variety of regions. There is little similarity between the pixels in low texture regions, but we can still estimate the image effectively. We can achieve flat surfaces without spots in flat regions and estimate the approximate contour in occluded regions. For the discontinuous region, we can estimate the texture of the object clearly.

## 5. Conclusions

We designed an efficient real-time end-to-end stereo matching network. In addition to the difficulties of occlusion areas, low textured regions, etc., the typical problem is the mutual exclusion of precision and speed. Three-dimensional convolution is beneficial to improve the effect, but it has more loss in speed. Therefore, we proposed our network to reduce the number of 3D convolutions. At the same time, we adopted lower resolution feature image input to reduce the computational amount to improve the speed. In order to maintain accuracy, we added a multi-cross attention module into the specific characteristic of the hourglass extractor and used a multi-level cost volume. Furthermore, the thin edge-guided network structure fills in the detailed information and improves the accuracy of the disparity regression. Compared with other networks, the speed and accuracy of our network are both higher, and the effectiveness of the network is proved by our experiments.

## Figures and Tables

**Table 1:** Multi-level cost volume ablation experiment on KITTI2015.

Model	Over-Fitting Location	EPE(%)		Avg Error Rate (Threshold = 3) (%)		Run-Time (s)
		All	Noc	All	Noc	
MCA Net-32	-	-	-	-	-	-
MCA Net-64	120	1.75	1.53	9.54	8.37	0.42
MCA Net-96	300	1.45	1.29	6.90	6.41	0.50
MCA Net-128	250	1.42	1.27	7.50	6.64	0.54

**Table 2:** Multi-cross attention module ablation experiment on KITTI2015.

Model	Over-Fitting Location	EPE (%)		Avg Error Rate (Threshold = 3) (%)		Run-Time (s)
		All	Noc	All	Noc	
MCA Net-cut	300	1.65	1.47	7.73	6.49	0.5049
MCA Net-cca	300	1.80	1.66	8.25	7.23	0.5132
MCA Net-mca	325	1.24	1.05	6.27	5.18	0.5050

**Table 3:** Test results on Scene Flow.

Model	Avg Error Rate (Threshold = 1) (%)	Num of 3D Conv Layers
PSM-Net [22]	12.10	25
GC-Net [21]	15.60	19
GA-Net-11 [12]	10.80	11
GA-Net-7 [12]	11.90	7
Our MCA Net	11.11	6
Stereo-Net [13]	41.12	4

**Table 4:** Test results on KITTI2012 and KITTI 2015.

Model	Num of 3D Conv Layers	Avg Error Rate (Threshold = 3) (%)				Run-Time(s)
		All		Noc		
		2012	2015	2012	2015	
MC-CNN [16]	-	3.63	3.88	2.43	3.33	67
GC-Net [21]	19	2.30	2.67	1.77	2.45	0.9
SSPCV-Net [29]	-	1.90	2.11	1.47	1.91	0.9
GA-Net-15 [12]	15	1.80	1.93	1.36	1.73	1.5
Our MCA Net	6	1.96	2.05	1.53	1.85	0.45
Stereo-Net [13]	4	6.02	4.83	4.91	4.30	0.015

## Data Availability

Not applicable.
